# Salivary Interleukin-6 and C-Reactive Protein/Mean Platelet Volume Ratio in the Diagnosis of Late-Onset Neonatal Pneumonia

**DOI:** 10.1155/2021/8495889

**Published:** 2021-10-18

**Authors:** Ahmed Omran, Yasmin Ali, Mohamed Osama Abdalla, Sonya El-Sharkawy, Ahmed R. Rezk, Abdelmoneim Khashana

**Affiliations:** ^1^Department of Pediatrics & Neonatology, Faculty of Medicine, Suez Canal University, Ismailia, Egypt; ^2^Department of Clinical Pathology, Faculty of Medicine, Suez Canal University, Ismailia, Egypt; ^3^Department of Pediatrics & Neonatology, Faculty of Medicine, Port Said University, Port Said, Egypt; ^4^Department of Pediatrics, Ain Shams University, Cairo, Egypt

## Abstract

Neonatal pneumonia is a serious respiratory infectious disease with a high rate of case fatality in developing countries. Salivary cytokines could serve as interesting noninvasive markers in the diagnosis of neonatal pneumonia. The aim was to assess the diagnostic role of salivary and serum interleukin-6 (IL-6), C-reactive protein/mean platelet volume (CRP/MPV) ratio, and the combination of these markers in the diagnosis of late-onset neonatal pneumonia in full-term neonates. Seventy full-term neonates, 35 with late-onset neonatal pneumonia and 35 controls, were enrolled in this prospective case-control study. Complete blood count (CBC), salivary and serum IL-6, and CRP concentrations were measured for all the study subjects. The sensitivity, specificity, positive predictive value, and negative predictive value of salivary IL-6, serum IL-6, and CRP/MPV ratio for the diagnosis of late-onset neonatal pneumonia were determined. At the cutoff point of >34 pg/ml, salivary IL-6 showed 82.86% sensitivity and 91.43% specificity. CRP/MPV ratio showed a sensitivity of 97.14% and specificity of 85.71% at a cutoff value > 0.88. The combination of salivary IL-6 and CRP/MPV ratio improved the sensitivity and specificity to 100%. The current study shows for the first time that both salivary IL-6 and CRP/MPV ratio are suitable markers for the diagnosis of late-onset neonatal pneumonia in full-term neonates.

## 1. Introduction

Neonatal pneumonia remains a major global health burden and contributes to annual deaths of nearly one million neonates that represent 10% of global child mortality [1]. Although diagnosis of neonatal pneumonia could be a challenging task, accurate and very early diagnosis is essential for proper treatment and avoids complications.

Interleukin-6 (IL-6) is a pleiotropic proinflammatory cytokine produced by a variety cells in response to infection [2]. Blood IL-6 is an early sensitive marker of neonatal bacterial infection and one of the most studied cytokines in the diagnosis of infection in neonates [3–12].

In early stages of pneumonia, alveolar macrophages produce proinflammatory cytokines. The main cytokines produced are tumor necrosis factor-*α* and IL-6, and their systemic and bronchoalveolar levels increase subsequently during the disease course [13, 14]. Blood IL-6 is a useful marker in determining the severity of the lung injury and inflammatory response in children with pneumonia, with higher levels correlating with disease severity and early mortality [15–17].

Saliva represents an excellent noninvasive, easy to perform, and patient-friendly biofluid for the screening of neonatal infections [18–20]. Salivary analysis is a potentially novel tool for the diagnosis of pneumonia in children [21, 22]. The salivary IL-6 level was significantly higher in children with pneumonia compared to healthy controls [22].

Blood C-reactive protein (CRP) level and mean platelet volume (MPV) can be used for the diagnosis of neonatal pneumonia [19]. MPV is a surrogate marker of platelet activation and is associated with neonatal infections and other inflammatory and infectious diseases [19, 20, 23–32]. In pediatrics, the CRP/MPV ratio can be used as a marker for the differential diagnosis between bacterial and viral pneumonia as well as the prediction of complications [33].

To the best of our knowledge, this is the first study to evaluate the applicability of salivary IL-6 and CRP/MPV ratio as diagnostic markers in full-term neonates with late-onset pneumonia.

## 2. Materials and Methods

### 2.1. Design and Patients

The present study is a prospective case-control study. Subjects included 35 full-term neonates diagnosed with late-onset pneumonia and 35 controls. The cases of the present study were recruited from the neonatal intensive care unit (NICU) of Suez Canal University Hospitals, Ismailia, Egypt, for the duration from January 2018 to January 2019. The protocol was approved by the Faculty of Medicine Institutional Review and Research Ethics Boards.

Inclusion criteria of cases were neonates with ≥37 weeks of gestational age, birth weight of ≥2.5 kg for both females and males, and age of 7 to 28 days of life admitted with clinical features of late-onset neonatal pneumonia. The definitive diagnosis of neonatal pneumonia was based on the presence of clinical manifestations in the form of various degrees of respiratory distress, intercostal or subcostal retractions, grunting, cough, and associated chest X-ray suggestive of pneumonia (includes lobar or segmental consolidation, diffuse haziness or granularity, nodular or coarse patchy infiltrates, and air bronchogram signs) [34].

Exclusion criteria were neonates with sepsis and other inflammatory conditions, preterm, low birth weight, major congenital anomalies, and antibiotics administration before admission.

The control group included matched age and sex neonates with no clinical features or risk factors for infection and followed up in our hospital for noninfectious neonatal jaundice.

### 2.2. Saliva Collection

We collected the saliva at 7 am, as salivary cytokines have independent diurnal rhythms [35]. Saliva collection was performed as mentioned in previously published work [19, 20, 36]. To avoid contamination by milk, samples were collected 1 hour before feeding. Collected samples were put immediately in polypropylene vials and stored at -20°C until batch analysis.

### 2.3. Laboratory Workup

Two ml of venous blood was collected and separated in plain and EDTA tubes. Complete blood count (CBC) was done with a Sysmex XN-550 Hematology Analyzer (Sysmex Corp., Kobe, Japan). To avoid false increase of the MPV value due to platelet swelling, CBC was done within less than 60 minutes of sampling. Serum CRP was quantified using the Cobas 6000 analyzer (Roche, Mannheim, Germany). The serum IL-6 level was assessed by an enzyme-linked immunosorbent assay (ELISA) commercial kit (ab46042, Abcam, UK). Salivary samples were centrifuged for 4000 rpm for 10 minutes. Supernatant was stored at-20°C. Salivary IL-6 was measured using a salivary IL-6 commercial ELISA kit (1-3602, Salimetrics, USA).

### 2.4. Statistical Analysis

The statistical analysis was done by the SPSS software, version 20 (SPSS, Chicago, IL, USA). To test the normality of data distribution, we used the Shapiro-Wilk test. For nonparametric quantitative data, differences between groups were assessed by Mann-Whitney *U*-test. For qualitative variables, testing significant differences was done by the chi-square test. Receiver operating characteristic (ROC) analysis was used to determine sensitivity, specificity, and optimal serum and salivary IL-6 cutoff values. A logistic regression model for each combination of biomarkers was performed with the late-onset neonatal pneumonia as the outcome. The predicted values of the logistic regression models were used to test for the predictive performance of the biomarker combination using ROC analysis. Significance is considered at *P* value of <0.05.

## 3. Results

### 3.1. Demographic Data

Thirty-five cases and 35 controls were included in the study. Males constitute 57.1% and 65.7% of cases and controls, respectively, with no statistically significant difference. The mean ages ± SD of cases and controls were 18 ± 6.9 and 19 ± 7.6 days, respectively, with no statistically significant difference. There were also no statistically significant differences between both groups (*P* value > 0.05) regarding gestational age, weight, mode of delivery, history of premature rapture of membrane (PROM), and maternal fever.

### 3.2. Clinical Data

Tachypnea and cough were the most significant presenting symptoms, 30 (85.7%) and 26 (74.3%), respectively, followed by fever 23 (65.7%). In chest examination, fine crepitation was found in 32 (91.4%), while diminished air entry was found in 24 (74.3%) and chest retraction in 25 (71.4%). Pneumonic patches and air bronchogram in chest X-ray were found in 24 cases (68.6%), and interstitial pneumonia was found in 11 cases (31.4%). Cases received oxygen support through the nasal cannula, nCPAP, and mechanical ventilation which were 16 (45.6%), 5 (14%), and 14 (40%), respectively, (Table 1).

### 3.3. Laboratory Data Findings

Total leukocytic count (TLC), platelet count, MPV, CRP, CRP/MPV ratio, and salivary and serum IL-6 values showed significant difference between the two groups. The pneumonia group showed higher TLC count and lower platelet count compared to the control group. CRP was significantly higher in the pneumonia group than the control group (mean 37.48 ± 32.72 and 3.29 ± 2.50 mg/l, respectively, *P* < 0.001). MPV was significantly different in the two groups (11.16 ± 1.49 and 9.77 ± 1.51 fl for cases and controls, respectively, *P* < 0.001). In the same context, the CRP/MPV ratio was significantly higher in cases than controls (mean 3.27 ± 2.52 and 0.36 ± 0.3, respectively, *P* < 0.001). Salivary IL-6 values were significantly different between the two groups (mean 140.37 ± 130.97 and 9.61 ± 13.72 pg/ml for cases and controls, respectively, *P* < 0.001). Serum IL-6 values showed also a significant difference between the groups (mean 167.86 ± 154.29 and 10.12 ± 11.10 pg/ml for cases and controls, respectively, *P* < 0.001) (Table 2).

### 3.4. Receiver Operating Characteristic (ROC) Analysis of the Tested Markers and Combination of Markers for the Diagnosis of Late-Onset Neonatal Pneumonia

**Figure 1:**
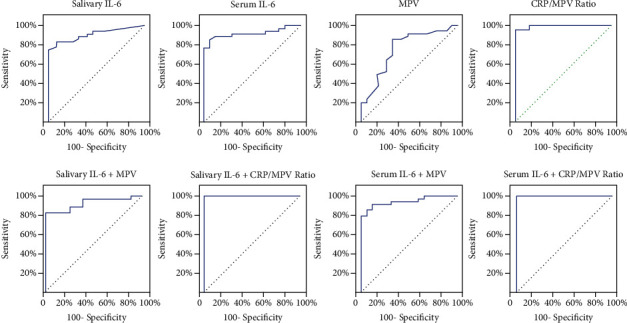
Receiver operator characteristics for the tested markers and their combination.

The ROC curve analysis for salivary and serum IL-6, MPV, and CRP/MPV ratio and for the combination of MPV with salivary and serum IL-6 and combination of CRP/MPV with salivary and serum IL-6 to detect late-onset neonatal pneumonia are shown in Figure 1.

For salivary IL-6, sensitivity was 82.86% and specificity was 91.43%, at a cutoff of 34 pg/ml. Serum IL-6 had a sensitivity and specificity of 88.5% at a cutoff of 15.3 pg/ml. MPV showed a sensitivity and specificity of 85.71% and 68.57%, respectively, at a cutoff value 9.7 fl. The CRP/MPV ratio showed a sensitivity and specificity of 97.14% and 85.71%, respectively, at a cutoff value 0.88 (Table 3). When combinations of different markers were used in the analysis, the diagnostic sensitivity and specificity were improved. We found that combining salivary or serum IL-6 with the CRP/MPV ratio markedly increased the sensitivity, specificity, PPV, and NPV to 100% (Table 4).

## 4. Discussion

Neonatal infection, including pneumonia, is one of the main causes of neonatal death worldwide with an estimation of responsibility of 23.4% of neonatal mortality each year [37]. Neonatal pneumonia is characterized by nonspecific clinical manifestations that are in common with other respiratory diseases which make it difficult to be identified and treated.

Saliva, as a biofluid, contains many of the same molecules present in the systemic circulation. Moreover, the concentration of most of the molecules present in the saliva is affected directly by their blood concentration. This makes saliva as a potentially useful diagnostic biofluid for disease detection and monitoring in neonates. Saliva has been studied as an alternative biological fluid suitable for a noninvasive diagnostic and follow-up tool in neonatal infections including salivary CRP in the diagnosis of neonatal sepsis and pneumonia [19, 20, 38–41] and screening of neonatal cytomegalovirus by salivary real-time PCR [42].

IL-6 is an early, highly sensitive proinflammatory cytokine and is one of the most widely studied cytokines in neonatal infections [9, 43–45]. IL-6 has a significant role in the early host response to infection, and its blood level changes precede that of CRP, which makes IL-6 an excellent parameter for the monitoring of neonatal sepsis [12].

In this study, the mean serum level of IL-6 was 167.86 ± 154.29 pg/ml in the case group, with sensitivity and specificity of 88.57% and 88.57%, respectively, at a cutoff value of 15.3 pg/ml for the detection of late-onset neonatal pneumonia. A previous cutoff value of 24.65 pg/ml for serum IL-6 was reported to detect neonatal sepsis with 72% sensitivity, 84% specificity, 95% PPV, and 42% NPV [21]. Other studies reported a wide range of different serum IL-6 cutoff levels (10-500 pg/ml). However, most of the reported cutoffs for the detection of neonatal sepsis fall between 10 and 30 pg/ml, which is in agreement with our results [46–48]. Qiu et al. investigated in their meta-analysis the diagnostic value of serum IL-6 as an early biomarker for neonatal sepsis with PROM. Their estimated sensitivity and specificity of serum IL-6 for the detection of neonatal sepsis with PROM were 87% and 88%, respectively, which was very close to our findings [11].

It was found that the serum IL-6 level had the highest efficacy for identifying bacterial pneumonia in children. It has 100% sensitivity and 99.14% specificity in discriminating bacterial pneumonia from viral and atypical pneumonia [49]. In addition, high IL-6 blood level is associated with increased mortality in less than 5-year-old patients with pneumonia requiring mechanical ventilation [50]. Moreover, in pediatric patient's community-acquired pneumonia, higher concentrations of circulating IL-6 has been correlated with worse outcomes and associated with early mortality [16].

Salivary biomarkers can be highly informative, especially in the early detection and discrimination of a variety of diseases and conditions in neonates, children, and adults [22, 51–54]. Salivary cytokines were detected within the first few hours after birth, and their levels decreased after 3 months [55]. Furthermore, salivary cytokines are promising potential biomarkers of bacterial infection in premature neonates [18].

To our knowledge, this is the first study to investigate the diagnostic value of salivary IL-6 as a noninvasive biomarker to detect late-onset pneumonia in full-term neonates. Salivary IL-6 values were found to be significantly higher in the case group compared to the control group (*P* < 0.001). Furthermore, we report a sensitivity of 82.86% and a specificity of 91.43% at cutoff point > 34 pg/ml of salivary IL-6. In a recent report, salivary IL-6 levels could predict bacterial infection in premature neonates with high sensitivity and specificity [18]. Klein et al. in 2104 reported that salivary analysis is a potentially useful diagnostic tool for pneumonia in the pediatric age group [56]. Very recently, Tsai and colleagues reported that salivary IL-6 and CRP levels are significantly higher in pediatric patients with pneumonia compared to healthy controls [22].

In our study, we investigated also for the first time the diagnostic value of the CRP/MPV ratio for the detection of late-onset neonatal pneumonia in full-term neonates. Here, we report a high ability of the CRP/MPV ratio to detect diseased neonates with sensitivity 97.14% and specificity of 85.71% at a cutoff value > 0.88. In children, the CRP/MPV ratio might be used in differentiating bacterial from viral pneumonia and prediction of complications [33].

The perfect diagnostic biomarker is a marker of approximately 100% sensitivity and 100% NPV. This can be attained by combining different markers for the diagnosis of neonatal infections including late-onset neonatal pneumonia. In this study, we found that combining of either serum or salivary IL-6 with the CRP/MPV ratio could detect neonatal pneumonia more effectively than each marker alone with 100% sensitivity and 100% NPV. Some previous studies investigated the diagnostic value of simultaneous combinations of several biomarkers in the diagnosis of neonatal infections. Combining CRP with IL-6 was more useful for the early detection of neonatal sepsis compared to each marker alone [4, 21]. Also, Zeitoun et al. showed that combining IL-10 with nCD64 improved the sensitivity and NPV for detecting of neonatal sepsis [57].

The main limitations of the current study are the small sample size and neonates with late-onset sepsis were only included.

## 5. Conclusion

This is the first study to report significantly higher salivary IL-6 and CRP/MPV ratio in neonates with late-onset pneumonia compared to controls. Combination of CRP/MPV with serum and salivary IL-6 improved the diagnostic accuracy of late-onset neonatal pneumonia in full-term neonates. Salivary IL-6 could be used as a potential noninvasive marker for the diagnosis of full-term neonates with late-onset neonatal pneumonia.

## Figures and Tables

**Table 1:** Clinical data, examination, and chest X-ray findings.

	Pneumonia (*n* = 35)		Control (*n* = 35)		*P* value
	No.	%	No.	%	
*Tachypnea*					
Absent	5	14.3	35	100.0	<0.001^∗^
Present	30	85.7	0	0.0	
*Cough*					
Absent	9	25.7	35	100.0	<0.001^∗^
Present	26	74.3	0	0.0	
*Fever*					
Absent	12	34.3	35	100.0	<0.001^∗^
Present	23	65.7	0	0.0	
*Retraction*					
Absent	10	28.6	35	100.0	<0.001^∗^
Present	25	71.4	0	0.0	
*Air entry*					
Normal	11	25.7	35	100.0	<0.001^∗^
Diminished	24	74.3	0	0.0	
*Oxygen supply*					
Room air	0	0.0	35	100	<0.001^∗^
Nasal cannula	16	45.7	0.0	0.0	
nCPAP	5	14.3	0.0	0.0	
Mechanical ventilation	14	40	0.0	0.0	
*CXR*					
Normal	0	0.0			
Pneumonic patches	24	68.6			
Interstitial pneumonia	11	31.4			

nCPAP: nasal continuous positive airway pressure; CXR: chest X-ray; ^∗^ indicates *P* < 0.05.

**Table 2:** Laboratory data in pneumonia and control groups.

	Pneumonia (*n* = 35) *mean* ± *SD*	Control (*n* = 35) *mean* ± *SD*	*P* value
Total leucocyte count (/mm^3^)	14.12 ± 6.56	8.91 ± 2.22	<0.001^∗^
Platelet count (10^3^/mm^3^)	172.83 ± 138.27	348.91 ± 131.54	<0.001^∗^
MPV (fl)	11.16 ± 1.49	9.77 ± 1.51	<0.001^∗^
CRP (mg/l)	37.48 ± 32.72	3.29 ± 2.50	<0.001^∗^
CRP/MPV ratio	3.27 ± 2.52	0.36 ± 0.30	<0.001^∗^
Salivary IL-6 (pg/ml)	140.37 ± 130.97	9.61 ± 13.72	<0.001^∗^
Serum IL-6 (pg/ml)	167.86 ± 154.29	10.12 ± 11.10	<0.001^∗^

MPV: mean platelet volume; CRP: C-reactive protein; IL-6: interleukin-6.

**Table 3:** Diagnostic performance of the tested markers for detection of late-onset neonatal pneumonia.

Parameter	AUC	95% CI	Cutoff value	Sensitivity (%)	Specificity (%)	PPV (%)	NPV (%)
Salivary IL-6 (pg/ml)	0.907	0.833–0.981	>34	82.86	91.43	90.6	84.2
Serum IL-6 (pg/ml)	0.921	0.848–0.993	>15.3	88.57	88.57	88.6	88.6
MPV(fl)	0.756	0.641–0.872	>9.7	85.71	68.57	73.2	82.8
CRP/MPV ratio	0.995	0.990–1.0	>0.88	97.14	85.71	87.2	96.8

IL-6: interleukin-6; MPV: mean platelet volume; CRP: C-reactive protein.

**Table 4:** Diagnostic performance of the tested marker combination for detection of late-onset neonatal pneumonia.

Parameter	AUC	95% CI	Sensitivity (%)	Specificity (%)	PPV (%)	NPV (%)
Salivary IL-6+ MPV	0.951	0.921-0.981	82.86	94.29	93.55	84.62
Salivary IL-6+ CRP/MPV	1.0	1.0-1.0	100	100	100	100
Serum IL-6+ MPV	0.952	0.928-0.976	82.86	94.29	93.55	84.62
Serum IL-6+ CRP/MPV	1.0	1.0-1.0	100	100	100	100

IL-6: interleukin-6; MPV: mean platelet volume; CRP: C-reactive protein.

## Data Availability

The data used to support the findings of this study are included within the article.
